# Comparative transcriptome and metabolome analyses reveal the methanol dissimilation pathway of *Pichia pastoris*

**DOI:** 10.1186/s12864-022-08592-8

**Published:** 2022-05-12

**Authors:** Yi-fan Yu, Jiashuo Yang, Fengguang Zhao, Ying Lin, Shuangyan Han

**Affiliations:** Guangdong Provincial Key Laboratory of Fermentation and Enzyme Engineering, School of Biology and Biological Engineering, South China University of Technology, Guangzhou, 510006 China

**Keywords:** Dissimilation pathway, Formaldehyde, Oxidative phosphorylation, Methanol metabolism, Glutathione

## Abstract

**Background:**

*Pichia pastoris* (*Komagataella phaffii*) is a model organism widely used for the recombinant expression of eukaryotic proteins, and it can metabolize methanol as its sole carbon and energy source. Methanol is oxidized to formaldehyde by alcohol oxidase (AOX). In the dissimilation pathway, formaldehyde is oxidized to CO_2_ by formaldehyde dehydrogenase (FLD), S-hydroxymethyl glutathione hydrolase (FGH) and formate dehydrogenase (FDH).

**Results:**

The transcriptome and metabolome of *P. pastoris* were determined under methanol cultivation when its dissimilation pathway cut off. Firstly, *Δfld* and *Δfgh* were significantly different compared to the wild type (GS115), with a 60.98% and 23.66% reduction in biomass, respectively. The differential metabolites between GS115 and *Δfld* were mainly enriched in ABC transporters, amino acid biosynthesis, and protein digestion and absorption. Secondly, comparative transcriptome between knockout and wild type strains showed that oxidative phosphorylation, glycolysis and the TCA cycle were downregulated, while alcohol metabolism, proteasomes, autophagy and peroxisomes were upregulated. Interestingly, the down-regulation of the oxidative phosphorylation pathway was positively correlated with the gene order of dissimilation pathway knockdown. In addition, there were significant differences in amino acid metabolism and glutathione redox cycling that raised our concerns about formaldehyde sorption in cells.

**Conclusions:**

This is the first time that integrity of dissimilation pathway analysis based on transcriptomics and metabolomics was carried out in *Pichia pastoris*. The blockage of dissimilation pathway significantly down-regulates the level of oxidative phosphorylation and weakens the methanol assimilation pathway to the point where deficiencies in energy supply and carbon fixation result in inefficient biomass accumulation and genetic replication. In addition, transcriptional upregulation of the proteasome and autophagy may be a stress response to resolve formaldehyde-induced DNA–protein crosslinking.

**Supplementary Information:**

The online version contains supplementary material available at 10.1186/s12864-022-08592-8.

## Background

The methylotrophic yeast *Pichia pastoris* (*Komagataella phaffii*) is one of most commonly used expression systems. It can metabolize methanol as its sole carbon and energy source [1–3]. Methanol is oxidized to formaldehyde by alcohol oxidase (AOX) in peroxisomes. Formaldehyde is further metabolized by assimilation or dissimilation. During assimilation, formaldehyde is directly fixed with dihydroxyacetone synthase (DAS) [4]. The entire methanol assimilation pathway is localized to peroxisomes. In the dissimilation pathway, formaldehyde is oxidized to S-formylglutathione by the NAD^+^-dependent enzyme formaldehyde dehydrogenase (FLD) in the first step, and S-formylglutathione then reacts spontaneously with glutathione to form S-hydroxymethyl glutathione. Then, S-formylglutathione hydrolase (FGH) hydrolase hydrolyses this compound into formate and glutathione. Next, formate is oxidized to CO_2_ by NAD + -dependent formate dehydrogenase (FDH) and released in vitro [5–8].

The dissimilation pathway metabolizes 2 molecules of NADH to the extent that it is considered to be the main source of power and energy. A surplus of FLD activity was shown to result in higher theoretical NADH formation rates and finally also in significantly improved butanediol production rates when a *P. pastoris* strain overexpressing FLD and butanediol dehydrogenase was applied for whole-cell biotransformation [9]. Additionally, by deleting the genes coding for dihydroxyacetone synthase isoforms 1 and 2 (DAS1 and DAS2), NADH regeneration via methanol oxidation (dissimilation) was increased significantly, which led to an increase in ATP and higher S-adenosylmethionine production [8, 10]. Therefore, an enhanced dissimilation pathway optimizes the energy distributions of methanol metabolism, guaranteeing more efficient NADH/product coupling and exploitation of both NADH steps for cofactor regeneration in whole-cell biotransformations [9].

Unfortunately, a carbon mass balance analysis revealed that 70–80% of the methanol metabolized is converted into carbon dioxide in the methanol fed-batch phase, when the methanol feed is in excess of the metabolic requirements in general [11–13]. This involves a huge loss of carbon atoms. Previous studies have reported the prevention of the conversion of methanol to CO_2_ via formaldehyde and formate by the knockout of genes for enzymes related to the dissimilation pathway. However, the *Δfld* strain suffered severe growth defects upon the addition of formaldehyde [14]. Similarly, whether in the methanol induction stage or the subsequent fermentation stage, the *Δfdh* strain showed a lower biomass and a slightly slower methanol consumption rate too [15]. Knockout studies on FLD and FDH of methylotrophic yeast demonstrated that the FLD knockout phenotype is more severe than the FDH knockout phenotype, which was explained by the higher toxicity of formaldehyde compared to formate [14]. Simply knocking out or weakening one or more genes in the dissimilation pathway without making up for the loss of NADH and ATP, not only results in the accumulation of toxic intermediates but also causes energy imbalance. It is instructive to determine the role of the dissimilation pathway before balancing carbon flow distribution and energy supply in the production and application of engineered yeast [15, 16].

The growth defect of methylotrophic yeast on high methanol medium is not caused directly by methanol toxicity but rather by formaldehyde, which is a key toxic intermediate of methanol metabolism [17]. As a strongly polarized reactive carbonyl compound, formaldehyde exists in various parts of the organism and plays an important role in cognitive ability and memory formation [18]. The toxicity of formaldehyde leads to partial synthetic lethality and growth defects in various cell lines utilizing methanol as a carbon source. Similarly, mice deficient in formaldehyde-metabolizing enzymes were found to develop partial synthetic lethality and mortality shortly after birth. This phenotype may be due to the accumulation of endogenous formaldehyde [19]. In fungi, plants or animals, the induction of DNA–protein crosslinking (DPC) formaldehyde is a topic of serious interest. Considering that DNA repair and RNA degradation pathways are evolutionarily conserved from yeast to humans, mechanisms of formaldehyde toxicity identified in yeast may be relevant to human disease and genetic susceptibility [20]. Formaldehyde dehydrogenase is critical to minimize the DPC issue, and gene-knockout organisms are commonly used to study the intracellular pathological changes of formaldehyde [19, 21, 22]. DPCs mainly consist of ribosomes and outer membrane proteins. Malfunction of these proteins may cause cell death because of outer membrane porin-induced programmed cell death or metabolic flux imbalance [23]. Membrane structure is a major target of methanol toxicity, while proteins are major targets of formaldehyde toxicity [24].

The dissimilation pathway is speculated to have two physiological functions: formaldehyde detoxification and energy production [4, 25, 26]. Based on these two points, we constructed three dissimilation pathway single gene-knockout strains by CRISPR/Cas9: a formaldehyde dehydrogenase-deficient strain (*∆fld*), an S-(hydroxymethyl) glutathione dehydrogenase-deficient strain (*∆fgh*) and a formate dehydrogenase-deficient strain (*∆fdh*). Then, we analysed key nodes and metabolic pathways by examining differentially expressed genes (DEGs) to discover the function of the dissimilation pathway and explore the stress response of yeast by comparing the transcriptome and metabolome of dissimilation pathway-defective strains and wild-type strains. Performing a whole-transcriptome analysis of dissimilation pathway knockout can promote the pathological study of formaldehyde metabolism and the development of industrial production strains.

## Results and discussion

### Phenotypic difference analysis

**Fig. 1:**
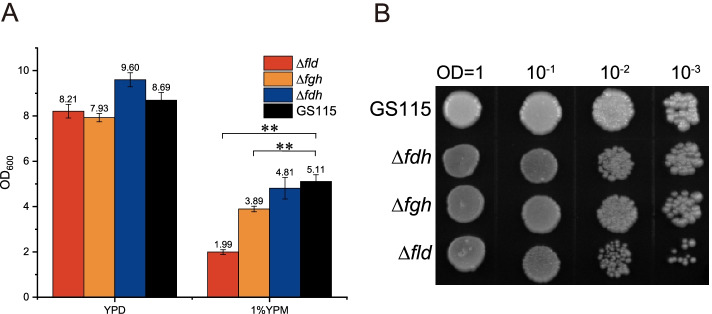
Phenotypic differences between knockout strains and wild strains. **A** The biomass difference between knockout strain and wild strain under the conditions of YPD or 1% YPM for 12 h. **p < 0.01 **B** the phenotypic difference between knockout strain and wild strain under 4% YPM plate culture condition for 7 days

We knocked out enzyme genes of the dissimilation pathway (FLD (PAS_chr3_1028), FGH (PAS_chr3_0867) and FDH (PAS_chr3_0932)) separately in GS115 by CRISPR/Cas9 and found no homologous sequences in the genome through BLAST [27]. With glucose as the main carbon source, there was no significant growth difference between dissimilation pathway knockout strains and GS115. However, with methanol as the main carbon source, the biomass of dissimilation pathway knockout strains (*∆fld, ∆fgh, ∆fdh*) was lower (60.98%, 23.66%, 5.69%) than that of wild-type strain (GS115) (Fig. 1. A). GS115 showed concordance with *∆fdh* and significant differences with *∆fld* and *∆fgh* at 0.01 level (*p* < 0.01) under YPM culture conditions. Therefore, the growth of GS115 was significantly higher than that of *∆fld* and *∆fgh* under methanol culture conditions. In addition, the 4% YPM plate used to monitor methanol tolerance between strains showed poor growth of dissimilation pathway knockout strains, particularly *∆fld* (Fig. 1. B).

### Comparative transcriptomic

**Fig. 2:**
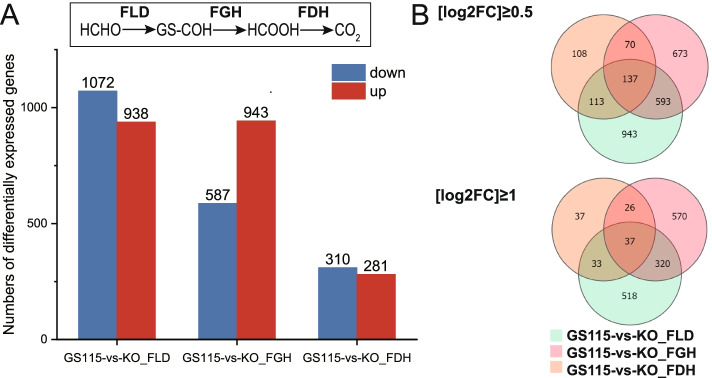
Comparative transcriptome. **A**. Number of DEGs between dissimilation pathway knockout and wild-type strains under methanol culture conditions (three parallel experiments). **B**. Venn diagrams of DEGs between dissimilation pathway knockout and wild-type strains under methanol culture conditions

Comparative transcriptomic were designed to reflect the effects of induced by gene knockout and methanol perturbations on metabolic pathways. The transcriptional profiles of *∆fld*, *∆fgh*, *∆fdh* and GS115 incubated in methanol for 12 h were developed and designated KO_FLD, KO_FGH, KO_FDH and GS115 respectively. GS115 and KO_FDH have the smallest sample variation. Then we compared the differences between GS115 versus KO_FLD (GL), GS115 versus KO_FGH (GG) and GS115 versus KO_FDH (GD). The upward and downward DEG numbers for GL, GG, and GD were 938 and 1072, 943 and 587, and 281 and 310, respectively (Fig. 2. A). The change in the number of down-regulated genes was positively correlated with the knockout order, which is consistent with the results of the biological phenotype, demonstrating that FLD is a key enzyme gene in the dissimilation pathway of methanol metabolism. Interestingly, single knockouts did not show significant differences in transcript levels of other genes in the dissimilation pathway. The DEGs of GL and GG were analyzed by transcription factor (TF) ranking (Supplemental Table 2). Among the up-regulated genes, the zinc cluster transcriptional activator (CAY71800, CAY69410) was enriched (*p* < 0.05); while among the down-regulated genes, carbon source-responsive zinc-finger transcription factor (CAY71743) and proposed transcriptional activator, member of the Gal4p family of zinc cluster proteins (CAY71429) was significantly enriched among the down-regulated genes (*p* < 0.01).

To understand the common impact of knockouts in the dissimilation pathway, we used Venn diagrams to show genes in different comparison groups. A total of 137 DEGs among the three groups were enriched in KEGG metabolic pathways (log2FC ≥ 0.5, q ≤ 0.05) (Fig. 2. B): glycolysis, TCA cycle, pentose and glucuronate interconversions, pyruvate metabolism biosynthesis of antibiotics, metabolism of various amino acids (tyrosine, phenylalanine, tryptophan, glycine, serine and threonine). A total of 357 DEGs among GL and GG were enriched in KEGG metabolic pathways (log2FC ≥ 1, q ≤ 0.05) (Fig. 2. B): most genes in the peroxisome pathway were transcriptionally upregulated, whereas a large proportion of genes in oxidative phosphorylation, glycolysis and TCA cycle were significantly downregulated. Furthermore, we were interested in genes involved in cysteine metabolism (*p* ≤ 0.05) and glutathione metabolism, related to formaldehyde binding.

### Comparative transcriptomics and metabolomics of ∆fld and wild-type strain

Formaldehyde dehydrogenase is the first enzyme in the dissimilation pathway, and its knockout can severely compromise strain robustness. We used BMM medium with methanol as the sole carbon source for 24 h to evaluate the metabolomic differences between *∆fld* and wild-type strain. All identified metabolites were classified: organic acids and derivatives (32.561%), lipids and lipid-like molecules (19.562%), and organoheterocyclic compounds (12.227%). The first three significantly abundant differential metabolic pathways were ABC transporters, amino acid biosynthesis, and protein digestion and absorption (Additional Fig. 1). Relatively in DEGs of GL, organonitrogen compound biosynthetic process, ribosome, cellular biosynthetic process, ATP metabolic process, peptide and oxoacid metabolic process were enriched for GO term; ribosome, Biosynthesis of antibiotics, glycolysis, biosynthesis of amino acids, oxidative phosphorylation, pentose and glucuronate interconversions were enriched for KEGG pathway.

**Fig. 3:**
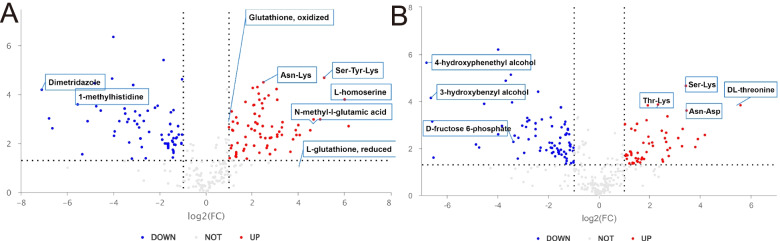
Analysis of differential metabolites between *Δfld* and GS115 in positive (**A**) and negative (**B**) ion mode

Based on univariate analysis, the differences among all metabolites (including unidentified metabolites) detected in positive and negative ion modes were analysed. In the positive ion mode (Fig. 3. A), oxidized glutathione (GSSG) (log2FC ≥ 1.17) and reduced glutathione (GSH) (log2FC ≥ 4.0) were upregulated; metabolites associated with glutathione availability were generally upregulated, such as N-methyl-L-glutamate (log2FC ≥ 4.6), homoserine (log2FC ≥ 6.0), His-Glu ( log2FC ≥ 2.8), and L-methionine (log2FC ≥ 1.7). Although the *Δfld* strain was unable to metabolise formylglutathione, the glutathione redox cycle was accelerated and may have reduced intracellular formaldehyde levels. Among the differential metabolites, other different species of amino acids were up-regulated, such as Ser-Tyr-Lys (log2FC ≥ 5.12), His-Asp (log2FC ≥ 2.0), Ile-Lys (log2FC ≥ 2.3), Val-Lys (log2FC ≥ 2.2), Asp-Leu (log2FC ≥ 4.0), Pro pro (log2FC ≥ 2.7), etc. And Ile-Pro was downregulated (log2FC ≤ -8.6). Among the differential metabolites, other different species of amino acids were up-regulated, such as Ser-Tyr-Lys (log2FC ≥ 5.12), His-Asp (log2FC ≥ 2.0), Ile-Lys (log2FC ≥ 2.3), Val-Lys (log2FC ≥ 2.2), Asp-Leu (log2FC ≥ 4.0), Pro-pro (log2FC ≥ 2.7), etc. In addition, acetyl coenzyme A was down-regulated (log2FC ≤ -1.4) and its branch metabolite N-acetyl histidine was significantly down-regulated (log2FC ≤ -5.1). However, diacetylchitosan was upregulated (log2FC ≥ 2.4).

In the negative ion mode (Fig. 3. B), the methanol assimilation pathway metabolites, D-glucose-6-phosphate (log2FC ≤ -1.3) and glyceraldehyde (log2FC ≤ -1.2) were down-regulated. Meanwhile, D-fructose (log2FC ≤ -1.5), D-gluconate (log2FC ≤ -1.7), glucitol (log2FC ≤ -1.8), and malate (log2FC ≤ -0.5) were down-regulated. D-ribose (log2FC ≤ -2.7) and thymine (log2FC ≤ -3.4) were down-regulated, indicating that *Δfld* is restricted during genetic replication, which may severely affect normal cell growth.

Through the interaction of DEGs of amino acid metabolism, aldehyde dehydrogenase (NAD +) (PAS_chr4_0043), glutamate dehydrogenase (NADP +) (PAS_chr1-1_0107), ornithine decarboxylase (PAS_chr3_0417) were screened out as important genes. The assimilation pathway and central carbon metabolites were down-regulated in *Δfld*, which may affect intracellular organic carbon fixation. The up-regulation of chitinose may be due to the resistance of chitin to oxidative stress. In contrast, most species of amino acids in the differential metabolites are up-regulated when synthesized from scratch. Therefore, knockdown of FLD, although not efficient in carbon utilization, facilitates amino acid metabolism and product synthesis.

### Oxidative phosphorylation

**Fig. 4:**
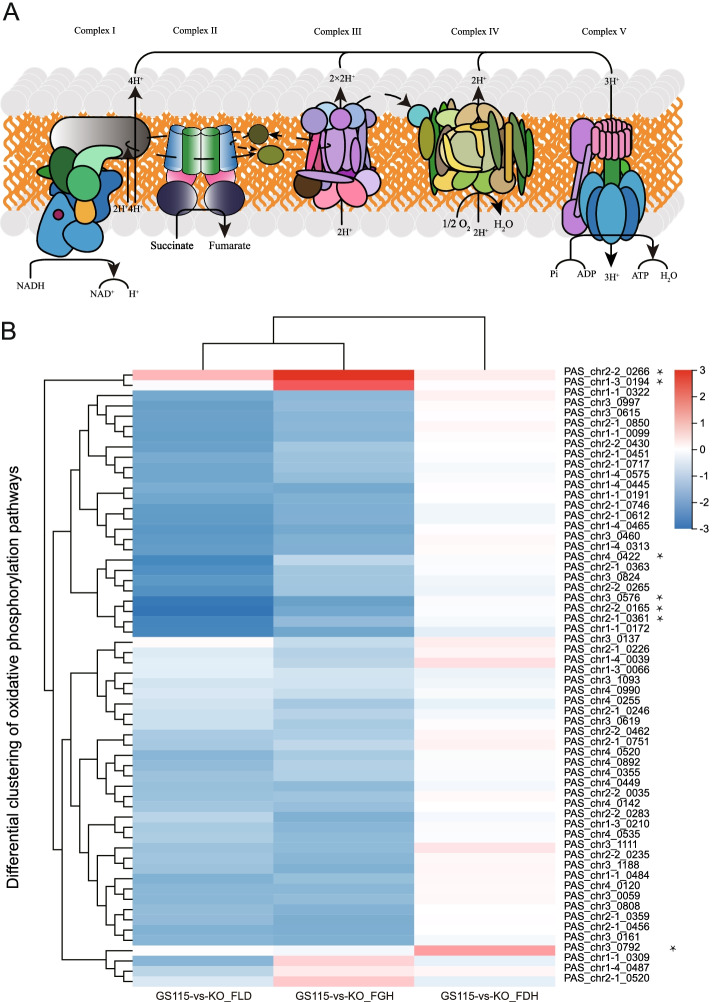
Oxidative phosphorylation pathway under methanol culture conditions. **A** Dialogue with oxidative phosphorylation in *Pichia pastoris* [28]. **B** DEGs of oxidative phosphorylation between dissimilation pathway knockout and wild-type strains

The 60 DEGs from the three groups in the oxidative phosphorylation pathways were clustered. The degree of oxidative phosphorylation of GD was similar, while GL and GG were significantly down-regulated (Fig. 4).

**Fig. 5:**
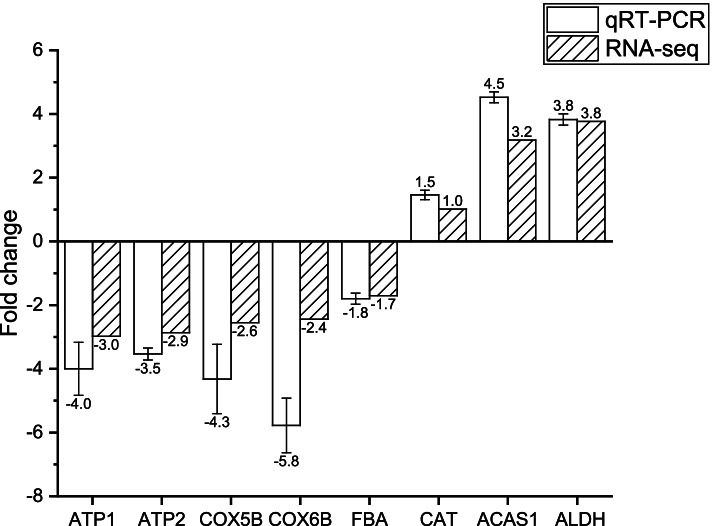
Fold change between the RNA-seq and qRT-PCR of DEGs

F-type H + -transporting ATPase subunit beta (ATP1 and ATP2, PAS_chr3_0576 and PAS_chr2-2_0165) were conspicuous downregulated in GL and GG (log2FC ≤ -1.5, q ≤ 0.05). The difference in fold change between RNA-seq and qRT-PCR of Oxidative phosphorylation is demonstrated in Fig. 5. ATP1 and ATP2 are proton-transporting ATP synthase complexes with nucleoside phosphatase activity that bind to purine nucleotides and act in nucleotide biosynthetic processes and nitrogen compound metabolism. In comparative metabolomics, differences in purine metabolism and pyrimidine metabolism of nucleotides may be associated with transcriptional down-regulation of mitochondria-related enzyme subunits. This leaves ATP in limited supply.

Cytochrome c oxidase subunit (COX5B and COX6B, PAS_chr2-1_0361 and PAS_chr4_0422) were downregulated in GL (log2FC ≤ -2.5, q ≤ 0.05) (Fig. 5). This severely diminished the mitochondrial-cytoplasmic proton transmembrane transporter protein activity. The transcript levels of the ubiquitin-cytochrome c reductase core subunit (e.g. UQCRC2, PAS_chr2-2_0430), which has catalytic activity for binding cations and metal ions, were down-regulated (log2FC ≤ -1.9, q ≤ 0.01), reducing the catalytic efficiency of the enzyme during oxidative phosphorylation. Transcript levels of NADH dehydrogenase complex subunits (e.g. NDUFB7, PAS_chr1-1_0172) were significantly down-regulated in GL (log2FC ≤ -2.4) and GG (log2FC ≤ -1.8, Q ≤ 0.02). It uses NAD(P)H, quinone or similar compounds as receptors and has oxidoreductase activity. Down-regulated genes in the oxidative phosphorylation pathway, such as the NADH dehydrogenase complex subunit, are associated with metabolic pathways in human Alzheimer's disease, cardiovascular disease and non-alcoholic fatty liver disease. The dissection of the mechanisms underlying strains defective in the dissimilatory pathway provides a theoretical basis for human pathology.

Screened for highly expressed genes (log2FC ≥ 2, q ≤ 0.05) in GL, revealed the upregulation of some dehydrogenase transcripts after FLD knockout, such as pyridoxine 4-dehydrogenase (PAS_chr4_0550), alcohol dehydrogenase (NADP +) (PAS_chr3_0006), NADPH2 dehydrogenase (PAS_chr3_1184), D-arabinose 1-dehydrogenase (PAS_chr2-1_0775), which may imply that other dehydrogenases compensate for the function of formaldehyde dehydrogenase (not shown in the picture).

In GG, cytochrome c oxidase subunit 7c (COX7C, PAS_chr2-2_0266) (log2FC ≥ 2.9, q ≤ 0.01) and haem o synthase (COX10, PAS_chr1-3_0194) (log2FC ≥ 2.0, q ≤ 0.01) were significantly upregulated. Others were downregulated. In GD, only NADH dehydrogenase (NDH, PAS_chr3_0792), a mitochondrial external NADH dehydrogenase or type II NAD(P)H: quinone oxidoreductase, was upregulated (log2FC ≥ 1.2, q ≤ 0.01). It might compensate for the absence of FDH.

### Methanol metabolism pathway

**Fig. 6:**
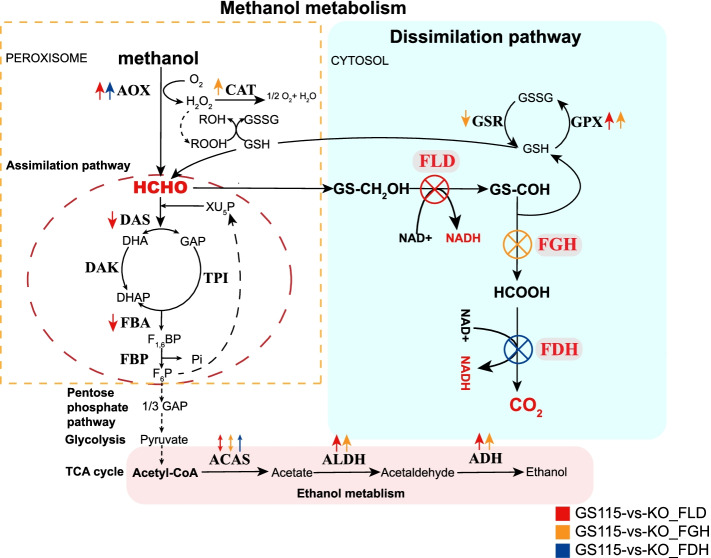
Methanol metabolism pathway and comparison of transcription levels in *Pichia pastoris*. The arrow indicates the comparative transcription level of the dissimilation pathway knockout and wild-type strains (q ≤ 0.05)

The main carbon metabolism pathways in *P. pastoris* include methanol metabolism, glycolysis, the TCA cycle, the pentose phosphate pathway and ethanol metabolism. By comparing transcriptomes, we attempted to explain the variation in the methanol metabolism pathway after knockout of the dissimilation pathway genes (Fig. 6). In peroxisomes, alcohol oxidase 2 (AOX2, PAS_chr4_0152) was upregulated in GL (log2FC ≥ 1.3, q ≤ 4.96E-12) and GD (log2FC ≥ 0.9, q ≤ 1E-04). AOX2 is induced by methanol, which may lead to an increase in formaldehyde content in the peroxisome.

Knockout of the dissimilation pathway gene down-regulates the assimilation pathway of formaldehyde. The dihydroxyacetone synthase (DAS, PAS_chr3_0832 and PAS_chr3_0834, log2FC ≤ -0.8) and fructose-bisphosphate aldolase (FBA, PAS_chr1-1_0072, log2FC ≤ -1.7) of the assimilation pathway were significantly downregulated when FLD was knocked out, which may be one of the reasons for the low biomass of *∆fld* (Fig. 5). Correspondingly, D-glucose 6-phosphate was downregulated (log2FC ≤ -1.3,VIP ≥ 1.8). This again validates the prominence of FLD in the dissimilation pathway.

When FGH was knocked out, catalase (CAT, PAS_chr2-2_0131, log2FC ≥ 1) was upregulated, which means reactive oxygen species (ROS) induced a pronounced oxidative stress response (Fig. 5). Superoxide dismutase [Cu–Zn] (PAS_chr4_0786), which destroys radicals normally produced within cells that are toxic to biological systems, was upregulated significantly in GL (log2FC ≥ 1.0, q ≤ 0.01) and GG (log2FC ≥ 2.5, q ≤ 0.01).

Previous studies have shown that under methanol culture conditions, genes encoding methanol metabolism are upregulated, and glycolysis and TCA cycle transcription are downregulated [29]. Significant transcriptional downregulation of genes involved in glycolysis and the TCA cycle was observed in GL and GG. Interestingly, malate dehydrogenase (MDH2, PAS_chr4_0815) was transcriptionally upregulated in GL, GG (log2FC ≥ 2.7, q ≤ 7.93E-06) and GD. Genes involved in alcohol metabolism were significantly transcriptionally upregulated (q ≤ 0.05). Among them, transcription of acetyl-coenzyme A synthetase (ACAS1, PAS_chr3_0403) and alcohol dehydrogenase (ADH, PAS_chr1-3_0153) were upregulated (log2FC ≥ 1, q ≤ 0.05) (Fig. 5). Aldehyde dehydrogenase (NAD +) (ALDH, PAS_chr3_0987) (log2FC ≥ 3.7, q ≤ 6.61E-07) was significantly upregulated in GG (Fig. 5). However, ACAS2 (PAS_chr2-1_0767) was downregulated in GL and GG (log2FC ≤ -1.4, q ≤ 6.61E-07). This may mean that the two ACASs are responsible for different metabolic pathways in *Pichia pastoris* and ACAS2 is more affected by methanol induction.

#### Glutathione redox cycling and amino acid metabolism

**Fig. 7:**
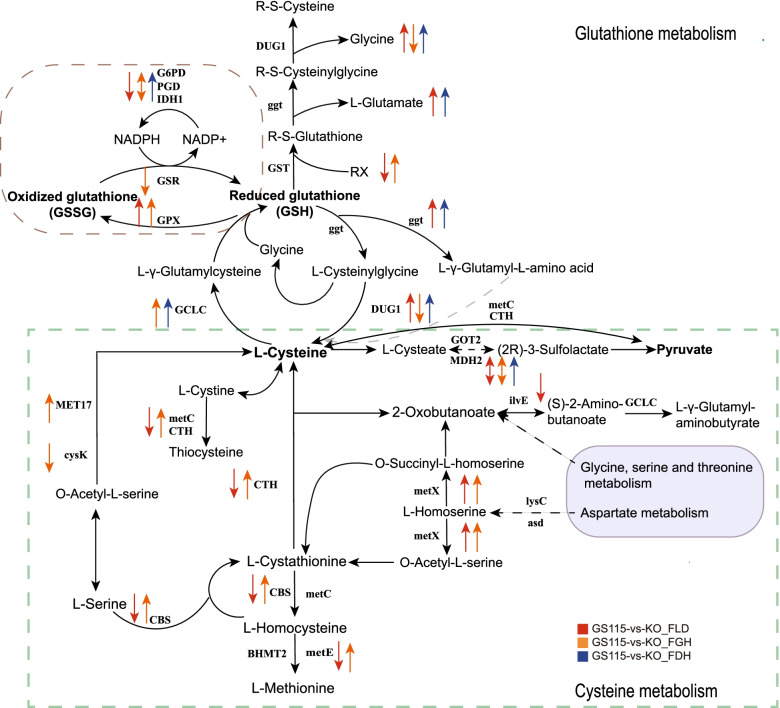
Comparative transcriptome analysis of glutathione and amino acid metabolism under methanol culture conditions [28]. The arrow indicates the comparative transcription level of the dissimilation pathway knockout and wild-type strains (q ≤ 0.05)

Glutathione (GSH, L-γ-glutamyl-L-cysteinylglycine) is the main sulfur compound and appears as the major nonprotein thiol compound in yeasts. In cells, glutathione mainly exists in the reduced form GSH, as oxidized glutathione (GSSG) is converted rapidly by glutathione reductase. In the process of glutathione reduction and oxidation (Fig. 7), glutathione peroxidase (GPX, PAS_chr2-2_0382) was upregulated (log2FC ≥ 0.6, q ≤ 0.05), while glutathione reductase (NADPH) (GSR, PAS_chr3_1011) was downregulated (log2FC ≤ -0.6, q ≤ 0.05) in GG. In the positive ion mode, GSH and GSSG were upregulated (log2FC ≥ 4.0 and log2FC ≥ 1.1, VIP ≥ 1) when FLD was knockout. Cystathionine gamma-lyase (CTH, PAS_chr1-4_0489) had zero expression in GS115_M. CTH, an enzyme involved in sulfur compound metabolism and cysteine metabolism, showed significantly upregulated transcription in GG (log2FC ≥ 10, q ≤ 2.7E-54).

We clustered the 149 DEGs in the amino acid metabolism. The knockout of each dissimilation gene produced a comparable up and down regulation trend, which may reveal the effect on the amino acid pathway such as arginine, proline, valine, leucine, isoleucine, lysine, glycine, serine and threonine metabolism. 5-Methyltetrahydropteroyltriglutamate-homocysteine methyltransferase (metE, PAS_chr2-1_0160) was upregulated in GG (log2FC ≥ 1.6, q ≤ 0.05). Proline dehydrogenase (PRODH, PAS_chr1-3_0269) is upregulated in order of knockout by sequence (log2FC ≥ 1.4, 2.8, 3.4, q ≤ 0.01), which facilitates the process of proline catabolism to glutamate. The glutamate-cysteine ligase catalytic subunit (GCLC, PAS_chr1-1_0184) is transcriptionally upregulated in GG and GD (log2FC ≥ 0.5, q ≤ 0.01). Glutathione S-transferase (GST, PAS_chr2-1_0490) was significantly upregulated in GG (log2FC ≥ 1.6, q ≤ 0.01) and downregulated in GL (log2FC ≤ -1.3, q ≤ 0.05). Cys-Gly metallodipeptidase (DUG1, PAS_chr3_0353) was significantly upregulated in GL (log2FC ≥ 2.1, q ≤ 1.5E-07). After FLD knockouted, L-homoserine was significantly upregulated (log2FC ≥ 6.0, VIP ≥ 4.9). Some amino acidsm, like Ser-Tyr-Lys, His-Asp, His-Glu, Ile-Lys, Val-Lys were up-regulated (VIP ≥ 1). And 1-methylhistidine was downregulated (log2FC ≤ -5.5, VIP ≥ 1). GO enrichment analysis revealed that in GL and GG, genes involved in the metabolic and biosynthetic processes of organic acids and carboxylic acids were transcriptionally downregulated, whereas in GD, they were transcriptionally upregulated (q ≤ 0.01). Another difference is that primary amine oxidase (AOC3, PAS_chr1-4_0441, PAS_chr2-1_0307 and PAS_chr4_0621) and amidase (amiE, PAS_chr3_0283) are upregulated in GL and GD. The knockout of two dehydrogenases in the dissimilation pathway may have affected the metabolism of organic nitrogen compounds.

### Upregulation of proteasomes, peroxisomes and autophagy

The knockout of genes in the dissimilation pathway caused upregulation of peroxisomes and autophagy. The transcription level of the DNA-dependent metalloprotease WSS1 (PAS_chr3_0200) increased significantly (log2FC ≥ 1.1(GG), log2FC ≥ 1.9(GL), q ≤ 0.01). The protein component of DPCs is targeted for repair by proteases of the Wss1/SPRTN family. This indicated that protease digestion of DPC was a stress response to formaldehyde. In GG, ubiquitin C (PAS_chr4_0762), AN1-type domain-containing protein (PAS_chr4_0567), 20S proteasome subunit alpha 2 (PAS_chr1-1_0433), which is in ubiquitin–proteasome pathway, was significantly upregulated; the difference was not significant in GD.

## Discussion

### Formaldehyde dehydrogenase is the key enzyme in the dissimilation pathway

Regardless of phenotypic differences or transcriptome differences, the results after FLD knockout are the most significant. FLD coordinates the formaldehyde level in methylotrophic strains according to the methanol concentration during growth [30]. After FLD is knocked out, the relevant enzyme genes of the assimilation pathway are downregulated, and the content of D-glucose 6-phosphate is decreasing. The assimilation and dissimilation of formaldehyde have a synergistic effect, and the down-regulation of the assimilation reduced biomass. Moreover, the difference in organic nitrogen metabolism also proves that FLD is a key enzyme that uses methanol and/or methylamine as carbon and/or nitrogen sources [16].

### Driving energy is the main function of the dissimilation pathway

Our transcripts validate that the level of oxidative phosphorylation is significantly down-regulated after FLD and FGH knocked out. Although knockout of FLD or FDH reduced NADP supply, the physiological role of FDH was revealed to be mainly detoxification of formate rather than stimulated energy generation [31]. The majority of methanol is metabolized via an energy-generating dissimilation pathway, leading to a corresponding increase in mitochondrial size and number [12]. FLD knockout significantly affected ATP production. FLD activity was identified as the main bottleneck of effective recovery of NADH through methanol dissimilation. In the engineered strain modification, butanediol productivity was improved by increasing FLD activity [9]. The energy driving effect of FLD and FGH on methanol metabolism is important for yeast biosynthesis.

### Adsorption of formaldehyde by glutathione and amino acids

GSH scavenges cytotoxic H_2_O_2_ and maintains a redox balance in the cellular compartments in the response of yeasts to different nutritional and oxidative stresses [32, 33]. GSH/GSSG ratio rose, suggesting stronger protection against oxidative stress, and was also correlated with high GSR activity [34, 35]. FGH is distributed between peroxisome and cytoplasm to release GSH in the dissimilation pathway [36]. An important reaction by formaldehyde is the formation of compounds with the tripeptide glutathione; between 50 and 80% of endogenous formaldehyde occurs in the form of compounds that include glutathione [16, 37]. Knockout of FLD increased the amount of GSSG and GSH compared with GS115. However down-regulation of GSH/GSSG ratio may increase intracellular oxidative stress. Interestingly, the up-regulation of CAT transcription confirms this when FGH was knockout so that GSH could not be restored. GPX transcription are upregulated in GL and GG. Knockout of FLD and FGH may accelerate the GSH redox cycle. This may mean that accelerated combination of glutathione and formaldehyde still cannot compensate for the impact of knocking out the dissimilation pathway.

The formation of S-[1-(N2-deoxyguanosinyl) methyl] glutathione between glutathione and DNA was induced by formaldehyde [32]. The involvement of the cysteine residue of GSH in coupling suggests that other thiols may participate in the formation of this type of DNA damage from formaldehyde [32]. The reactions of formaldehyde with cysteine and histidine are alternative routes of formaldehyde detoxification metabolism [33]. The up-regulated amino acid metabolites were detected after FLD knockouted. In the context of the upregulation of many amino acid biosynthesis genes or proteins, this suggests an increased flux towards amino acid and protein synthesis [38]. Knockout of the dissimilation pathway caused differences in amino acid metabolism, but the internal associations and regular pattern need to be explored. The exploration of amino acid metabolism and methanol metabolism or formaldehyde dehydrogenase knockout samples is of great significance for revealing the toxicity of formaldehyde and physiological mechanism.

### DPC caused by formaldehyde during methanol metabolism

Formaldehyde is the key intermediate of methanol detoxification metabolism and the origin metabolite of the dissimilation pathway. We speculate that the upregulation of peroxisomes accelerates the exchange of formaldehyde, what is more, upregulation of proteasome and autophagy transcription may be to solve the DPC caused by formaldehyde.

In eukaryotes, the autophagy–lysosome system and the ubiquitin–proteasome system (UPS) are the two major quality control pathways responsible for maintaining proteome homeostasis and directing recycling to meet nutrient demand. Formaldehyde exposure triggers widespread ubiquitylation events in cells [39, 40]. The polymerization of ubiquitin, a key molecule known to work in concert with the proteasome, served as a degradation signal for numerous target proteins [41, 42]. Proteasome cleaved the protein moiety of DPC to remove the lesion. Additionally, when FLD is knocked out, differential metabolites are enriched in purine and pyrimidine metabolism. Nucleotide excision repair and homologous recombination are involved in repairing DPCs [43].

In contrast, autophagy can eliminate larger protein complexes, insoluble protein aggregates, and even entire organelles and pathogens in toto due to the sheer size of the engulfing autophagic vesicles [41]. Methanol oxidation stress results in the accumulation of hydrogen peroxide and organic injury [44, 45]. Excessive ROS are formed from peroxisome metabolism when methanol-grown wild-type cells are exposed to excess methanol [46]. The cut-off of the dissimilation pathway may not be able to carry out effective methanol metabolism and thus aggravate the accumulation of ROS. The upregulation of the autophagy pathway may be related to the degradation of damaged peroxisomes or other components of eukaryotic cells [29, 47].

Mutations in the gene encoding the putative human homologue of a yeast DPC protease cause premature ageing and cancer predisposition syndrome in humans [48]. In *Saccharomyces cerevisiae*, several genes involved in DNA repair (eight RAD genes) that have been identified as specific for methanol toxicity were previously reported as determinants of tolerance for formaldehyde [49]. The up-regulation of DNA repair genes may be caused by the accumulation of formaldehyde by knocking out FLD and FGH. It is also of interest that genes related to DPCs in *P. pastoris* could be mining. However, it is also a challenge to determine the concentration of formaldehyde in peroxisomes of yeast.

## Conclusions

The main function of the dissimilation pathway may be the supply of energy rather than formaldehyde detoxification. Knockout of FLD and FGH significantly downregulated oxidative phosphorylation, leaving the strains with inadequate energy supply and low biomass. Knockdown of the dissimilation pathway leads to downregulation of gene expression in glycolysis and the TCA cycle. In particular, the assimilation pathway was downregulated. Differences in amino acid and glutathione metabolism were apparent, probably due to the adsorption of amino acid and glutathione to formaldehyde to resolve formaldehyde accumulation and oxidative stress responses. In addition, knockout of the dissimilation pathway enhanced the stress response to formaldehyde. Upregulation of the proteasome, peroxisome and autophagy may be the solution of DPC induced by formaldehyde.

## Methods

### CRISPR/Cas9

*P. pastoris* GS115 was used as the host for CRISPR/Cas9-based genome editing studies. Efficient CRISPR/Cas9-mediated genome editing with a type III promoter (i.e., SER promoter) to drive the expression of the single guide RNA (sgRNA) was achieved [50]. Using CRISPRdirect (http://crispr.dbcls.jp/) to design a targeting sequence (Table 1), a CRISPR plasmid vector (Cas9-III promoter-targeting sequence-gRNA) was successfully constructed [51]. The donor was synthesized with the upper and lower 1000 bp of the target gene (Table 2). Donor and Cas9 plasmids were transformed into Pichia pastoris for expression (100 μg/mL ampicillin supplemented when necessary). The successful knockout strains were screened and cultured in YPD for a period of time to remove the plasmids. Single colonies were picked and crushed in a metal bath at 80 °C in yeast crushing solution. After a short centrifugation, 1 μL of supernatant was taken as PCR template and PCR identification was performed using xxx-DONOR-F and xxx-DONOR-R. The PCR products of wild strain GS115 were 3085 bp (FDH), 2866 bp (FGH) and 3007 bp (FLD) in length. The successful knockout resulted in product fragment lengths of 2040 bp (*Δfdh*), 2019 bp (*Δfgh*) and 1806 bp (*Δfld*).

**Table 1:** PAM site of dissimilation pathway knockout gene

Target genes	PAM (5’-3’)
FLD	CGGAAACGAGAAGTCCCGTC
FGH	AACCCCACTAAAGCCCCATG
FDH	GAGGTTACGGTGGTGACGTC

**Table 2:** Donor primers for PCR

Donor primers	sequence(5’-3’)
FLD-DONOR-F	GCTGTCAGTTCTGCGTCAACATGGGC
FLD-donor-1-R	GTACGACGTATGATGAATGAATGAGTTATGTAAGGCC
FLD-donor-2-F	CTCATTCATTCATCATACGTCGTACCTTCCCTCCCA
FLD-DONOR-R	GCCGCAACTTTAATTATCCGTGGATTGGGAGCT
FGH-DONOR-F	CACCATACAGGTCTCCCTTCGATACCAGTGCAAAGT
FGH-dornor-1-R	TGAATGTAGAAAGATGGAATGAACGCTACAGCGAGGAAG
FGH-donor-2-F	TCCATCTTTCTACATTCACAGACGTTCATGCTGCTC
FGH-DONOR-R	CTTCTAGTCCCCCATTTGTGGCTTACGTAA
FDH-DONOR-F	AAATGGCAGAAGGATCAGCCTGGACGAAGCAACCAG
FDH-donor-1-R	TACCGTTCATGTTTAAGTGGGTGATGTTGGAGG
FDH-donor-2-F	CACTTAAACATGAACGGTAAGTACAAGACCAAGGC
FDH-donor-R	GCCTCAACAATTGGCAGCTCTTCTACGGT

### Strains and cultivation

*P. pastoris* GS115 (Invitrogen, USA), a histidine auxotrophic strain, was used as the host strain for the construction of recombinant strains [52]. Methylotrophic yeast grows well in a liquid medium containing 1% (vol/vol) methanol as the carbon source to induce the production of heterologous proteins. Culture conditions for transcriptome analysis: the strains were grown on YPD agar medium containing 2% agar, 1% yeast extract, 2% peptone and 2% glucose to obtain the seed liquid. In YP liquid medium (1% yeast extract, 2% peptone) containing 1% (vol/vol) methanol (YPM medium) or 2% glucose (YPD medium), the cells were cultured for 12 h to the exponential logarithmic phase of growth [53]. Culture conditions for metabolome analysis were as follows: in BMM liquid medium containing 100 mM potassium phosphate (pH 6.0), 1.34% YNB, 4 × 10–5% biotin and 0.5% methanol, the cells were cultured for 24 h.

### Methanol tolerance plate test

The strains were cultured in YPD for 24 h. The seed liquid was diluted to OD_600_ of 1 with YP liquid medium and diluted to different times in 96-well plates. 3 μl of diluent is used for spotting in 4% YPM plate and incubate for 7 days.

### Transcriptome and metabolome sample testing

The cultured cells were immediately frozen in a liquid nitrogen tank, and the samples were collected and sent to BGI Genomics Co.Ltd (Wuhan, China) for RNA extraction and transcriptome sequencing, and Shanghai Applied Protein Technology Co.Ltd (Shanghai, China) for metabolite group detection. Data are presented as the mean of three replicates ± standard deviation.

### Transcriptome data analysis

The data was tested on the DNBSEQ platform. The sequencing data was filtered with SOAPnuke (v1.5.2) by (1) Removing reads containing sequencing adapter; (2) Removing reads whose low-quality base ratio (base quality less than or equal to 5) is more than 20%; (3) Removing reads whose unknown base (N' base) ratio is more than 5%, afterwards clean reads were obtained and stored in FASTQ format [54]. The clean reads were mapped to the reference genome using HISAT2 (v2.0.4) [55]. Bowtie2 (v2.2.5) was applied to align the clean reads to the reference coding gene set, then expression level of gene was calculated by RSEM (v1.2.12) [56]. The heatmap was drawn by pheatmap (v1.0.8) according to the gene expression in different samples. Essentially, differential expression analysis was performed using the DESeq2 (v1.4.5) with Q value ≤ 0.05 [57, 58]. To take insight to the change of phenotype, GO (http://www.geneontology.org) and KEGG (https://www.kegg.ip/) enrichment analysis of annotated different expressed gene was performed by Phyper (https://en.wikipedia.org/wiki/Hypergeometric distribution) based on Hypergeometrictest. The significant levels of terms and pathways were corrected by Q value with a rigorous threshold (Q value ≤ 0.05) by Bonferroni. Rank Genes by TF were analysed on N.C. Yeastract *K.phaffii* (http://yeastract-plus.org/ncyeastract/kphaffii/formrankbytf.php) [59, 60].

### Metabolome data analysis

In this experiment, the metabolic profile changes of the samples were analysed by a metabonomics method based on UHPLC-Q-TOFMS technology. Metabonomics usually takes strict OPLS-DA VIP > 1 and P value < 0.05 as the screening criteria for significantly different metabolites. Before the annotation and analysis of KEGG pathways, the differential metabolites screened by positive and negative ion patterns were combined. The significant levels of metabolite enrichment in each pathway were analysed and calculated by Fisher’s accurate test to determine the metabolic and signal transduction pathways that were significantly affected [61].

### qRT-PCR

After extraction using the hot acidic phenol method, total RNA was treated with RNase-free DNase I (Thermo Scientific) for 42 °C, 5 min according to the manufacturer’s protocol. Subsequently, first-strand cDNA synthesis and qPCR were performed using the RevertAid First Strand cDNA Synthesis Kit (Thermo Scientific) and the PowerUp™ SYBR™ Green Master Mix (Thermo Scientific). For the negative control, the reverse transcriptase was omitted in the cDNA synthesis reaction with the DNase-treated total RNA as the template. No PCR product was obtained using the negative control as the template, suggesting that the genomic DNA was removed completed [53]. Data are presented as the mean of six replicates ± standard deviation.

The relative amount of fold change in gene expression was calculated using the comparative Ct method as follows.

Log2^−[(Ct value of the target gene of the sample to be tested − Ct value of the internal reference gene of the sample to be tested) − (Ct value of the target gene of the control sample − Ct value of the internal reference gene of the control sample)]^.

## Supplementary Information

**Additional file 1: Figure 1.** KEGG clustering analysis of differential metabolites between formaldehyde dehydroge-nase knockout strains and wild strains. Each bubble in the bubble plot represents a metabolic pathway (the top 20 most significant were selected based on *P*-value).

**Additional file 2: Figure 2.** Transcription-related DEGs in P. pastoris. Red represents significant up-regulation and blue represents significant down-regulation, with shades of colour indicating the degree of up- and down-regulation.

**Additional file 3: Supplemental table 1.** List of DEGs encoding transcripition factors in *p. pastoris.*

**Additional file 4: Supplemental table 2.** TF rank of the DEGs in *p.pastoris.*

## Data Availability

The RNA sequence datasets generated and/or analysed during the current study are available in the [NCBI Sequence Read Archive (SRA)] repository, [https://www.ncbi.nlm.nih.gov/biosample/]. Our recent submission to the BioSample database has been successfully processed and will be released on the date specified. BioSample accessions: SAMN20981851, SAMN20981852, SAMN20981853, SAMN20981854. Temporary Submission ID: SUB10230863. Release date: 2022–09-08, or with the release of linked data, whichever is first. All metabolomics data generated or analysed during this study are included in this published article [and its supplementary information files].

## Abbreviations

AOX: Alcohol Oxidase
FLD: Formaldehyde Dehydrogenase
FGH: S-Hydroxymethyl Glutathione Hydrolase
FDH: Formate Dehydrogenase
DPCs: DNA-Protein Crosslinks
DAS: Dihydroxyacetone Synthase
ACAS: Acetyl-Coenzyme A Synthetase
ADH: Alcohol Dehydrogenase
ALDH: Aldehyde Dehydrogenase
FBA: Fructose-Bisphosphate Aldolase
CAT: Catalase
GPX: Glutathione Peroxidase
GSR: Glutathione Reductase
COX7C: Cytochrome C Oxidase Subunit 7c
COX10: Haem O Synthase
ROS: Reactive Oxygen Species
GSH: Reduced Glutathione
GSSG: Oxidized Glutathione
metE: 5-Methyltetrahydropteroyltriglutamate-Homocysteine Methyltransferase
CTH: Cystathionine Gamma-lyase
AOC: Amine Oxidase
amiE: Amidase
PRODH: Proline Dehydrogenase
GCLC: Glutamate-Cysteine Ligase Catalytic Subunit
GST: Glutathione S-transferase
DUG: Cys-Gly Metallodipeptidase
UPS: Ubiquitin–Proteasome System
