# Supplementary material for: Comparative transcriptome and metabolome analyses reveal the methanol dissimilation pathway of Pichia pastoris
Source: BMC Genomics. 2022 May 12;23:366. doi: 10.1186/s12864-022-08592-8 (PMC9103059; doi:10.1186/s12864-022-08592-8)
Supplement: Supplementary file 1 — Additional file 1: Figure 1. KEGG clustering analysis of differential metabolites between formaldehyde dehydroge-nase knockout strains and wild strains. Each bubble in the bubble plot represents a metabolic pathway (the top 20 most significant were selected based on P-value). [file 12864_2022_8592_MOESM1_ESM.pdf]

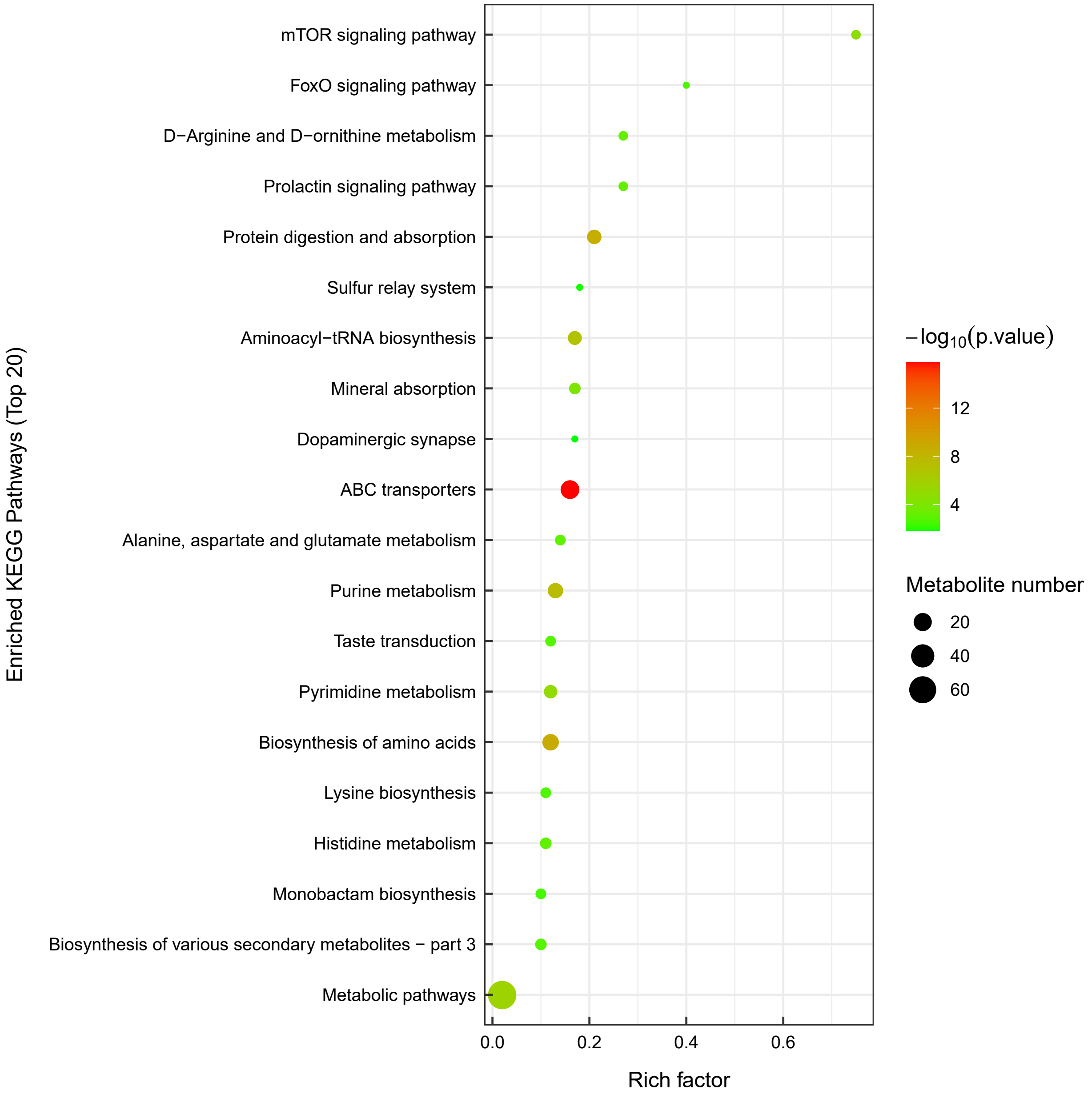

Additional Figure 1. KEGG clustering analysis of differential metabolites between formaldehyde dehydrogenase knockout strains and wild strains. Each bubble in the bubble plot represents a metabolic pathway (the top 20 most significant were selected based on P-value).
