# Supplementary material for: Comparative transcriptome and metabolome analyses reveal the methanol dissimilation pathway of Pichia pastoris
Source: BMC Genomics. 2022 May 12;23:366. doi: 10.1186/s12864-022-08592-8 (PMC9103059; doi:10.1186/s12864-022-08592-8)
Supplement: Supplementary file 2 — Additional file 2: Figure 2. Transcription-related DEGs in P. pastoris. Red represents significant up-regulation and blue represents significant down-regulation, with shades of colour indicating the degree of up- and down-regulation. [file 12864_2022_8592_MOESM2_ESM.pdf]

DEGs related to transcripition

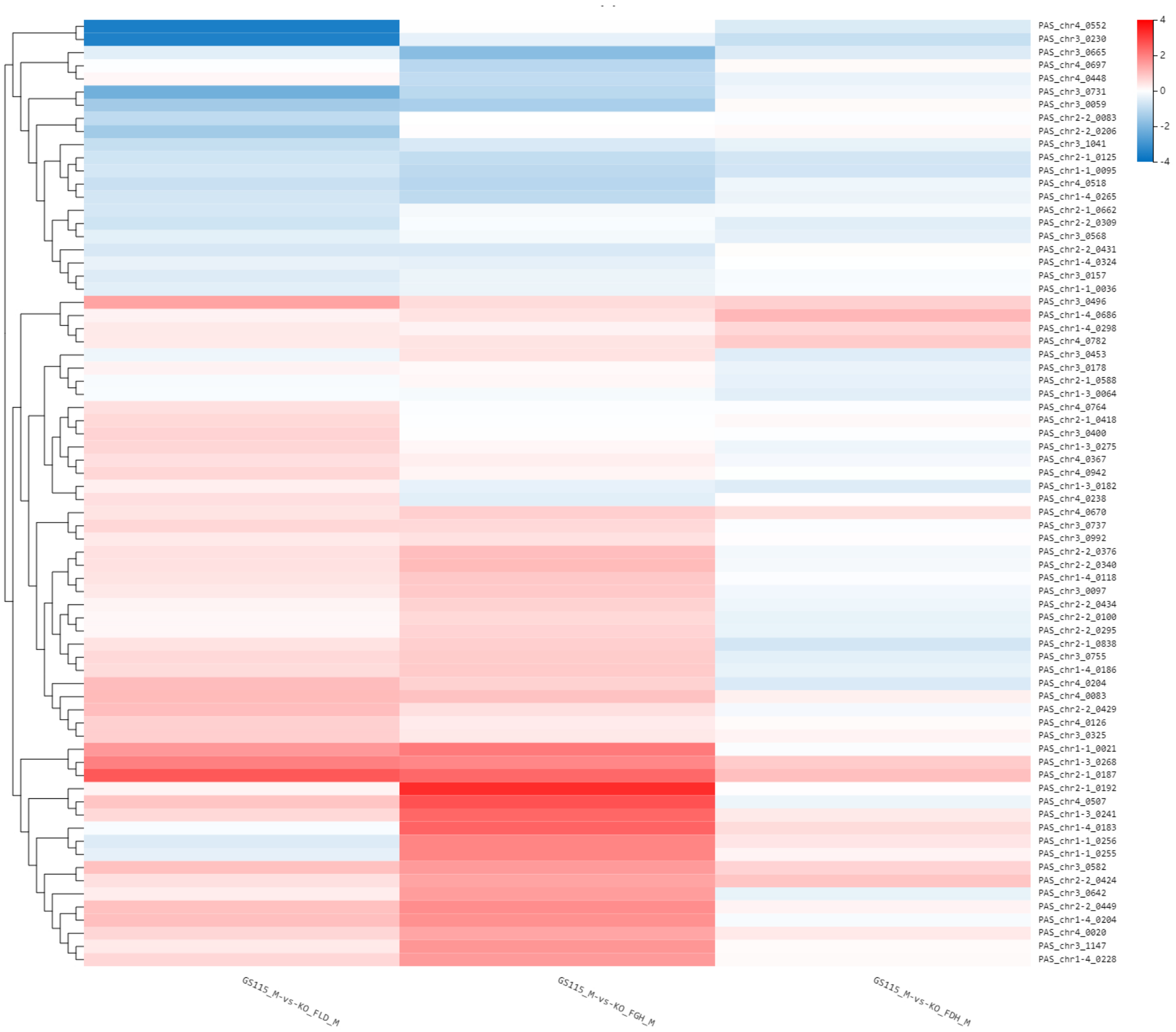

Additional Figure 2. transcription-related DEGs in *P. pastoris*. Red represents significant up-regulation and blue represents significant down-regulation, with shades of colour indicating the degree of up- and down-regulation.
