# Supplementary material for: Comparative transcriptome and metabolome analyses reveal the methanol dissimilation pathway of Pichia pastoris
Source: BMC Genomics. 2022 May 12;23:366. doi: 10.1186/s12864-022-08592-8 (PMC9103059; doi:10.1186/s12864-022-08592-8)
Supplement: Supplementary file 3 — Additional file 3: Supplemental table 1. List of DEGs encoding transcripition factors in p. pastoris. [file 12864_2022_8592_MOESM3_ESM.docx]

**Supplemental table 1 List of DEGs encoding transcripition factors in *p. pastoris***

| Gene ID | log2(GL) | Qvalue(GL) | log2(GG) | Qvalue(GG) | log2(GD) | Qvalue(GD) | PPI Protein ID | Nr |
| --- | --- | --- | --- | --- | --- | --- | --- | --- |
| PAS_chr1-1_0021 | 1.6174 | 0.0000 | 2.0939 | 0.0000 | -0.0831 | 0.9320 | 4922.CAY67375 | XP_002489656.1\|2.5e-140\|Hypothetical protein PAS_chr1-1_0021 [Komagataella phaffii GS115] |
| PAS_chr1-1_0036 | -0.4361 | 0.0181 | -0.3031 | 0.3833 | -0.0935 | 0.9167 | 4922.CAY67394 | XP_002489675.1\|1.2e-296\|Hsp70 protein that interacts with Zuo1p (a DnaJ homolog) to form a ribosome-associated complex [Komagataella phaffii GS115] |
| PAS_chr1-1_0095 | -0.6794 | 0.0000 | -1.0562 | 0.0082 | -0.7273 | 0.0000 | 4922.CAY67456 | XP_002489737.1\|2.3e-273\|Subunit (60 kDa) of TFIID and SAGA complexes [Komagataella phaffii GS115] |
| PAS_chr1-1_0255 | -0.4100 | 0.0025 | 1.9127 | 0.0020 | 0.1833 | 0.7705 | 4922.CAY67625 | XP_002489906.1\|0.0e+00\|Subunit of the THO complex [Komagataella phaffii GS115] |
| PAS_chr1-1_0256 | -0.5486 | 0.0028 | 1.9219 | 0.0008 | 0.4055 | 0.3080 | 4922.CAY67626 | XP_002489907.1\|6.3e-44\|Hypothetical protein PAS_chr1-1_0256 [Komagataella phaffii GS115] |
| PAS_chr1-3_0064 | -0.0928 | 0.4866 | -0.1532 | 0.6319 | -0.4406 | 0.0022 | 4922.CAY67116 | XP_002489400.1\|0.0e+00\|Hypothetical protein PAS_chr1-3_0064 [Komagataella phaffii GS115] |
| PAS_chr1-3_0182 | 0.2467 | 0.2168 | -0.3957 | 0.4463 | -0.5283 | 0.0242 | 4922.CAY67245 | XP_002489526.1\|0.0e+00\|Essential splicing factor [Komagataella phaffii GS115] |
| PAS_chr1-3_0241 | 0.6096 | 0.0010 | 2.3873 | 0.0016 | 0.3274 | 0.1180 | 4922.CAY67303 | XP_002489584.1\|1.5e-123\|Hypothetical protein PAS_chr1-3_0241 [Komagataella phaffii GS115] |
| PAS_chr1-3_0268 | 1.9940 | 0.0000 | 1.8823 | 0.0020 | 0.8140 | 0.1369 | 4922.CAY67332 | XP_002489613.1\|5.9e-114\|Hypothetical protein PAS_chr1-3_0268 [Komagataella phaffii GS115] |
| PAS_chr1-3_0275 | 0.6467 | 0.0009 | 0.1256 | 0.6443 | -0.2728 | 0.5003 | 4922.CAY67340 | XP_002489621.1\|0.0e+00\|Hypothetical protein PAS_chr1-3_0275 [Komagataella phaffii GS115] |
| PAS_chr1-4_0118 | 0.4243 | 0.0022 | 0.8589 | 0.0375 | -0.0510 | 0.9328 | 4922.CAY67947 | XP_002490228.1\|0.0e+00\|DEAH-box RNA-dependent ATPase/ATP-dependent RNA helicase [Komagataella phaffii GS115] |
| PAS_chr1-4_0183 | -0.0950 | 0.7931 | 2.4521 | 0.0000 | 0.5425 | 0.1557 | 4922.CAY68016 | XP_002490297.1\|2.2e-131\|TATA-binding protein, general transcription factor [Komagataella phaffii GS115] |
| PAS_chr1-4_0186 | 0.5543 | 0.0001 | 0.8336 | 0.0139 | -0.3557 | 0.1784 | 4922.CAY68019 | XP_002490300.1\|1.3e-270\|U1 snRNP protein involved in splicing [Komagataella phaffii GS115] |
| PAS_chr1-4_0204 | 1.0015 | 0.0014 | 1.7299 | 0.0017 | -0.1043 | 0.8232 | 4922.CAY68037 | XP_002490318.1\|0.0e+00\|Component of the holoenzyme form of RNA polymerase transcription factor TFIIH [Komagataella phaffii GS115] |
| PAS_chr1-4_0228 | 0.6308 | 0.0000 | 1.5869 | 0.0023 | 0.0789 | 0.8519 | 4922.CAY68065 | XP_002490344.1\|2.6e-64\|Core Sm protein Sm D2 [Komagataella phaffii GS115] |
| PAS_chr1-4_0265 | -0.6981 | 0.0027 | -1.0367 | 0.0309 | -0.2958 | 0.4212 | 4922.CAY68099 | XP_002490380.1\|6.0e-137\|Hypothetical protein PAS_chr1-4_0265 [Komagataella phaffii GS115] |
| PAS_chr1-4_0298 | 0.3279 | 0.0922 | 0.2095 | 0.4230 | 0.6335 | 0.0006 | 4922.CAY68138 | XP_002490419.1\|1.7e-226\|Mitochondrially localized type 2C protein phosphatase [Komagataella phaffii GS115] |
| PAS_chr1-4_0324 | -0.3525 | 0.0063 | -0.4081 | 0.4376 | -0.0089 | 0.9863 | 4924.XP_001384965.2 | XP_002490446.1\|0.0e+00\|RNA polymerase I subunit A135 [Komagataella phaffii GS115] |
| PAS_chr1-4_0686 | 0.1986 | 0.6849 | 0.4410 | 0.1877 | 1.1343 | 0.0000 | 4922.CAY68379 | XP_002490659.1\|1.3e-78\|Small nuclear ribonucleoprotein-associated protein [Komagataella phaffii GS115] |
| PAS_chr2-1_0125 | -0.7378 | 0.0000 | -0.9466 | 0.0319 | -0.6958 | 0.0000 | 4922.CAY68731 | XP_002491011.1\|0.0e+00\|RNA polymerase II second largest subunit B150, part of central core [Komagataella phaffii GS115] |
| PAS_chr2-1_0187 | 2.6138 | 0.0002 | 2.3620 | 0.0013 | 1.0061 | 0.0872 | 4922.CAY68795 | XP_002491075.1\|8.1e-139\|Hypothetical protein PAS_chr2-1_0187 [Komagataella phaffii GS115] |
| PAS_chr2-1_0192 | 0.1838 | 0.8381 | 3.3125 | 0.0000 | 0.0325 | 0.9841 | 4922.CAY68800 | XP_002491080.1\|0.0e+00\|Protein of unknown function [Komagataella phaffii GS115] |
| PAS_chr2-1_0418 | 0.6165 | 0.0308 | -0.0264 | 0.9258 | 0.1091 | 0.8908 | 1005962.W1QEG5 | OUM51684.1\|0.0e+00\|hypothetical protein BVG19_g804 [[Candida] boidinii] |
| PAS_chr2-1_0588 | -0.1165 | 0.5398 | 0.1253 | 0.5761 | -0.3872 | 0.0178 | 44689.DDB0266833 | XP_002491504.1\|0.0e+00\|Subunit (61/68 kDa) of TFIID and SAGA complexes [Komagataella phaffii GS115] |
| PAS_chr2-1_0662 | -0.6652 | 0.0001 | -0.1617 | 0.4877 | -0.1330 | 0.6471 | 4922.CAY69307 | XP_002491587.1\|0.0e+00\|Large subunit of the nuclear mRNA cap-binding protein complex [Komagataella phaffii GS115] |
| PAS_chr2-1_0838 | 0.4362 | 0.0352 | 0.7529 | 0.0020 | -0.7014 | 0.0008 | 4922.CAY69061 | XP_002491341.1\|5.9e-296\|hypothetical protein PAS_chr2-1_0838 [Komagataella phaffii GS115] |
| PAS_chr2-2_0083 | -1.0172 | 0.0132 | -0.0037 | 0.9946 | -0.0699 | 0.9551 | 4922.CAY69846 | XP_002492126.1\|1.4e-92\|Hypothetical protein PAS_chr2-2_0083 [Komagataella phaffii GS115] |
| PAS_chr2-2_0100 | 0.1213 | 0.5050 | 0.6095 | 0.0595 | -0.3694 | 0.0001 | 4922.CAY69827 | XP_002492107.1\|0.0e+00\|GTPase component of U5 snRNP involved in mRNA splicing via spliceosome [Komagataella phaffii GS115] |
| PAS_chr2-2_0206 | -1.4606 | 0.0000 | 0.0299 | 0.9330 | 0.0882 | 0.9373 | 44689.DDB0205029 | XP_002491992.1\|5.1e-89\|Hypothetical protein PAS_chr2-2_0206 [Komagataella phaffii GS115] |
| PAS_chr2-2_0295 | 0.1317 | 0.3080 | 0.6861 | 0.0357 | -0.3394 | 0.0013 | 4922.CAY69613 | XP_002491893.1\|0.0e+00\|TFIID subunit (150 kDa), involved in RNA polymerase II transcription initiation [Komagataella phaffii GS115] |
| PAS_chr2-2_0309 | -0.7628 | 0.0030 | -0.0926 | 0.8033 | -0.4722 | 0.1984 | 4922.CAY69597 | XP_002491877.1\|4.5e-76\|RNA polymerase subunit ABC14.5, common to RNA polymerases I, II, and III [Komagataella phaffii GS115] |
| PAS_chr2-2_0340 | 0.4673 | 0.0599 | 1.0753 | 0.0462 | -0.1612 | 0.7909 | 4922.CAY69566 | XP_002491846.1\|7.7e-260\|Conserved zinc-finger domain protein involved in pre-mRNA splicing [Komagataella phaffii GS115] |
| PAS_chr2-2_0376 | 0.4734 | 0.0127 | 1.0403 | 0.0364 | -0.1958 | 0.5741 | 4922.CAY69529 | XP_002491809.1\|1.9e-132\|Hypothetical protein PAS_chr2-2_0376 [Komagataella phaffii GS115] |
| PAS_chr2-2_0424 | 0.4798 | 0.2932 | 1.4492 | 0.0126 | 0.9173 | 0.0978 | 4922.CAY69485 | XP_002491765.1\|8.4e-248\|Splicing factor, component of the U4/U6-U5 snRNP complex [Komagataella phaffii GS115] |
| PAS_chr2-2_0429 | 1.0330 | 0.0008 | 0.4841 | 0.3264 | -0.1570 | 0.8418 | 4922.CAY69480 | XP_002491760.1\|2.2e-44\|Core Sm protein Sm F [Komagataella phaffii GS115] |
| PAS_chr2-2_0431 | -0.6049 | 0.0007 | -0.5992 | 0.0614 | 0.0448 | 0.9459 | 4922.CAY69478 | XP_002491758.1\|0.0e+00\|RNA polymerase III subunit C82 [Komagataella phaffii GS115] |
| PAS_chr2-2_0434 | 0.1647 | 0.6712 | 0.6987 | 0.0167 | -0.2453 | 0.6969 | 4922.CAY69475 | XP_002491755.1\|2.4e-83\|RNA polymerase subunit ABC23, common to RNA polymerases I, II, and III [Komagataella phaffii GS115] |
| PAS_chr2-2_0449 | 0.9811 | 0.0000 | 1.7886 | 0.0012 | 0.1868 | 0.7166 | 4922.CAY69909 | XP_002492189.1\|3.2e-169\|hypothetical protein PAS_chr2-2_0449 [Komagataella phaffii GS115] |
| PAS_chr3_0059 | -1.4458 | 0.0054 | -1.3476 | 0.0598 | 0.0748 | 0.9560 | 4922.CAY69987 | XP_002492267.1\|9.3e-52\|Subunit f of the F0 sector of mitochondrial F1F0 ATP synthase, which is a large, evolutionarily cons [Komagataella phaffii GS115] |
| PAS_chr3_0097 | 0.3534 | 0.3865 | 0.8470 | 0.0377 | -0.2156 | 0.7665 | 4922.CAY70026 | XP_002492306.1\|2.4e-124\|Nuclear protein that binds to RNA and to Mex67p, required for export of poly(A)+ mRNA from the nucle [Komagataella phaffii GS115] |
| PAS_chr3_0157 | -0.5501 | 0.0065 | -0.2973 | 0.6141 | -0.1248 | 0.8442 | 340170.XP_007375817.1 | XP_002492363.1\|4.1e-118\|RNA polymerase subunit ABC27, common to RNA polymerases I, II, and III [Komagataella phaffii GS115] |
| PAS_chr3_0178 | 0.1975 | 0.3709 | 0.0876 | 0.6696 | -0.3332 | 0.0383 | 42374.XP_002417572.1 | XP_002492387.1\|4.3e-222\|TFIIE large subunit [Komagataella phaffii GS115] |
| PAS_chr3_0230 | -3.5185 | 0.0000 | -0.3960 | 0.0985 | -0.8890 | 0.0000 | 33169.AAS52868 | XP_002492443.1\|0.0e+00\|ATPase involved in protein folding and the response to stress [Komagataella phaffii GS115] |
| PAS_chr3_0325 | 0.7626 | 0.0000 | 0.3582 | 0.0484 | 0.1913 | 0.2822 | 4922.CAY70363 | XP_002492542.1\|0.0e+00\|Essential splicesome assembly factor [Komagataella phaffii GS115] |
| PAS_chr3_0400 | 0.6861 | 0.0277 | -0.0223 | 0.9451 | -0.0289 | 0.9729 | 4922.CAY70449 | XP_002492628.1\|1.2e-196\|Hypothetical protein PAS_chr3_0400 [Komagataella phaffii GS115] |
| PAS_chr3_0453 | -0.2450 | 0.1265 | 0.4339 | 0.0520 | -0.5129 | 0.0008 | 4922.CAY70500 | XP_002492679.1\|0.0e+00\|Subunit (90 kDa) of TFIID and SAGA complexes [Komagataella phaffii GS115] |
| PAS_chr3_0496 | 1.4480 | 0.0000 | 0.5565 | 0.0143 | 0.7406 | 0.0153 | 4922.CAY70544 | XP_002492723.1\|0.0e+00\|RNA-dependent ATPase in the DEAH-box family [Komagataella phaffii GS115] |
| PAS_chr3_0568 | -0.4304 | 0.0340 | -0.1784 | 0.5384 | -0.4117 | 0.0453 | 4922.CAY70616 | XP_002492795.1\|0.0e+00\|RNA polymerase II largest subunit B220 [Komagataella phaffii GS115] |
| PAS_chr3_0582 | 0.9948 | 0.0410 | 1.5809 | 0.0054 | 0.6945 | 0.1071 | 4922.CAY70629 | XP_002492808.1\|2.6e-60\|Zinc cluster protein involved in pre-mRNA splicing and cycloheximide resistance [Komagataella phaffii GS115] |
| PAS_chr3_0642 | 0.2944 | 0.5234 | 1.5604 | 0.0012 | -0.3611 | 0.2238 | 4922.CAY70691 | XP_002492870.1\|4.3e-299\|Subunit of the SF3a splicing factor complex, required for spliceosome assembly [Komagataella phaffii GS115] |
| PAS_chr3_0665 | -0.4396 | 0.4029 | -1.8515 | 0.0004 | -0.5519 | 0.2070 | 4922.CAY70712 | XP_002492891.1\|8.0e-81\|17-kDa component of the U4/U6aU5 tri-snRNP, plays an essential role in pre-mRNA splicing [Komagataella phaffii GS115] |
| PAS_chr3_0731 | -2.2126 | 0.0000 | -1.0717 | 0.2183 | -0.2415 | 0.8306 | 1005962.W1QJL2 | XP_018208630.1\|0.0e+00\|heat shock protein SSB1 [Ogataea polymorpha] |
| PAS_chr3_0737 | 0.6231 | 0.0047 | 0.6028 | 0.0121 | -0.0663 | 0.8475 | 4922.CAY70787 | XP_002492966.1\|2.2e-156\|TFIIE small subunit, involved in RNA polymerase II transcription initiation [Komagataella phaffii GS115] |
| PAS_chr3_0755 | 0.5892 | 0.0001 | 0.7936 | 0.0259 | -0.4270 | 0.0242 | 4922.CAY70805 | XP_002492984.1\|2.5e-250\|Splicing factor, component of the U4/U6-U5 snRNP complex [Komagataella phaffii GS115] |
| PAS_chr3_0992 | 0.3431 | 0.2018 | 0.4696 | 0.0481 | 0.0360 | 0.9446 | 4922.CAY71054 | XP_002493233.1\|0.0e+00\|Protein involved in pre-mRNA splicing [Komagataella phaffii GS115] |
| PAS_chr3_1041 | -0.9036 | 0.0002 | -0.5958 | 0.3346 | -0.3706 | 0.5187 | 4922.CAY71104 | XP_002493283.1\|1.5e-62\|small nucleolar ribonucleoprotein SNU13 [Komagataella phaffii GS115] |
| PAS_chr3_1147 | 0.3499 | 0.3180 | 1.6478 | 0.0112 | 0.0658 | 0.9459 | 4922.CAY69971 | XP_002492251.1\|1.1e-269\|hypothetical protein PAS_chr3_1147 [Komagataella phaffii GS115] |
| PAS_chr4_0020 | 0.6355 | 0.1004 | 1.4153 | 0.0335 | 0.3511 | 0.5963 | 4922.CAY71246 | XP_002493425.1\|4.7e-137\|Component of the spliceosome complex involved in pre-mRNA splicing [Komagataella phaffii GS115] |
| PAS_chr4_0083 | 1.0785 | 0.0000 | 0.9607 | 0.0003 | 0.2306 | 0.5813 | 4922.CAY71309 | XP_002493488.1\|3.1e-264\|Splicing factor that is found in the Cef1p subcomplex of the spliceosome [Komagataella phaffii GS115] |
| PAS_chr4_0126 | 0.7441 | 0.0000 | 0.3206 | 0.2837 | 0.0657 | 0.9039 | 4922.CAY71353 | XP_002493532.1\|0.0e+00\|Hypothetical protein PAS_chr4_0126 [Komagataella phaffii GS115] |
| PAS_chr4_0204 | 1.0526 | 0.0001 | 0.7086 | 0.0000 | -0.5839 | 0.0465 | 4922.CAY71430 | XP_002493609.1\|3.2e-183\|Subunit of TFIIH and nucleotide excision repair factor 3 complexes [Komagataella phaffii GS115] |
| PAS_chr4_0238 | 0.5096 | 0.0205 | -0.4379 | 0.2443 | 0.0371 | 0.9341 | 4922.CAY71467 | XP_002493646.1\|0.0e+00\|Subunit of TFIIH and nucleotide excision repair factor 3 complexes [Komagataella phaffii GS115] |
| PAS_chr4_0367 | 0.5034 | 0.0093 | 0.2437 | 0.3369 | -0.1587 | 0.7421 | 4922.CAY71610 | XP_002493789.1\|2.6e-131\|Hypothetical protein PAS_chr4_0367 [Komagataella phaffii GS115] |
| PAS_chr4_0448 | 0.1194 | 0.8387 | -0.9707 | 0.0070 | -0.3287 | 0.4919 | 4922.CAY71701 | XP_002493880.1\|2.3e-50\|Lsm (Like Sm) protein [Komagataella phaffii GS115] |
| PAS_chr4_0507 | 0.9290 | 0.0005 | 2.7248 | 0.0000 | -0.2875 | 0.5211 | 4922.CAY71763 | XP_002493942.1\|4.1e-253\|Hypothetical protein PAS_chr4_0507 [Komagataella phaffii GS115] |
| PAS_chr4_0518 | -0.8661 | 0.0000 | -1.1099 | 0.0175 | -0.2633 | 0.1270 | 4922.CAY71773 | XP_002493952.1\|2.1e-304\|TFIIF (Transcription Factor II) largest subunit [Komagataella phaffii GS115] |
| PAS_chr4_0552 | -3.5716 | 0.0000 | -0.0142 | 0.9729 | -0.5665 | 0.0666 | 4929.XP_001482859.1 | ODV95030.1\|0.0e+00\|hypothetical protein PACTADRAFT_43220 [Pachysolen tannophilus NRRL Y-2460] |
| PAS_chr4_0670 | 0.4199 | 0.1544 | 0.7727 | 0.0242 | 0.5251 | 0.0415 | 4922.CAY71929 | XP_002494108.1\|4.8e-102\|Hypothetical protein PAS_chr4_0670 [Komagataella phaffii GS115] |
| PAS_chr4_0697 | -0.0241 | 0.9617 | -1.1055 | 0.0179 | 0.0815 | 0.9328 | 4922.CAY71959 | XP_002494138.1\|4.3e-180\|RNA polymerase III subunit C34 [Komagataella phaffii GS115] |
| PAS_chr4_0764 | 0.4793 | 0.0001 | -0.0694 | 0.8749 | -0.0451 | 0.9044 | 4922.CAY72028 | XP_002494207.1\|2.9e-265\|Splicing factor associated with the spliceosome [Komagataella phaffii GS115] |
| PAS_chr4_0782 | 0.3415 | 0.1171 | 0.4287 | 0.4028 | 0.8335 | 0.0000 | 4922.CAY72048 | XP_002494227.1\|1.2e-111\|Hypothetical protein PAS_chr4_0782 [Komagataella phaffii GS115] |
| PAS_chr4_0942 | 0.6245 | 0.0210 | 0.1513 | 0.5176 | -0.0368 | 0.9381 | 4922.CAY71540 | XP_002493719.1\|4.2e-291\|hypothetical protein PAS_chr4_0942 [Komagataella phaffii GS115] |
