# Supplementary material for: Comparative transcriptome and metabolome analyses reveal the methanol dissimilation pathway of Pichia pastoris
Source: BMC Genomics. 2022 May 12;23:366. doi: 10.1186/s12864-022-08592-8 (PMC9103059; doi:10.1186/s12864-022-08592-8)
Supplement: Supplementary file 4 — Additional file 4: Supplemental table 2. TF rank of the DEGs in p.pastoris. [file 12864_2022_8592_MOESM4_ESM.docx]

**Supplemental table 2 TF rank of the DEGs in *p. pastoris***

| DEGs | Transcription Factor |  | % in user set | % in *kphaffii* | p-value |
| --- | --- | --- | --- | --- | --- |
| UP (GL) | CAY70887 | Hypothetical protein | 34.58% | 20.01% | 0.023473 |
|  | CAY71800 | Zinc cluster transcriptional activator | 0.32% | 42.86% | 0.025152 |
|  | CAY69410 | Zinc cluster transcriptional activator | 0.21% | 40.00% | 0.046661 |
| DOWN (GL) | CAY68570 | Lactose regulatory protein | 14.76% | 30.37% | 3.59E-08 |
|  | CAY71743 | Carbon source-responsive zinc-finger transcription factor | 50.66% | 23.12% | 0.000213 |
|  | CAY71429 | Proposed transcriptional activator, member of the Gal4p family of zinc cluster proteins | 19.17% | 24.23% | 0.005117 |
|  | CAY68204 | Hypothetical protein | 1.97% | 30.43% | 0.02163 |
| UP (GG) | CAY68204 | Hypothetical protein | 2.34% | 31.88% | 0.002232 |
|  | CAY70887 | Hypothetical protein | 35.00% | 20.32% | 0.010889 |
|  | CAY71800 | Zinc cluster transcriptional activator | 0.32% | 42.86% | 0.025434 |
|  | CAY69410 | Zinc cluster transcriptional activator | 0.21% | 40.00% | 0.047063 |
|  | CAY70604 | Zinc-finger protein involved in transcriptional control of both nuclear and mitochondrial genes | 1.91% | 25.71% | 0.047742 |
| DOWN (GG) | CAY71429 | Proposed transcriptional activator, member of the Gal4p family of zinc cluster proteins | 21.64% | 15.08% | 0.000275 |
|  | CAY71743 | Carbon source-responsive zinc-finger transcription factor | 51.96% | 13.08% | 0.000776 |
|  | CAY71926 | Hypothetical protein | 20.95% | 14.15% | 0.004287 |
|  | CAY68570 | Lactose regulatory protein | 12.78% | 14.51% | 0.012977 |
| UP (GD) | CAY71743 | Carbon source-responsive zinc-finger transcription factor | 53.23% | 7.08% | 0.00326 |
|  | CAY71004 | Hypothetical protein | 0.65% | 20.00% | 0.019617 |
|  | CAY69410 | Zinc cluster transcriptional activator | 0.32% | 20.00% | 0.03286 |
|  | CAY68691 | tRNA methyltransferase, localizes to both the nucleus and mitochondrion to produce the modified base | 0.32% | 16.67% | 0.047326 |
|  | CAY69995 | Peptide methionine sulfoxide reductase, reverses the oxidation of methionine residues | 0.32% | 16.67% | 0.047326 |
| DOWN (GD) | CAY68204 | Hypothetical protein | 3.56% | 14.49% | 0.001261 |
|  | CAY69410 | Zinc cluster transcriptional activator | 0.36% | 20.00% | 0.02731 |
|  | CAY70604 | Zinc-finger protein involved in transcriptional control of both nuclear and mitochondrial genes | 2.49% | 10.00% | 0.038154 |
|  | CAY68691 | tRNA methyltransferase, localizes to both the nucleus and mitochondrion to produce the modified base | 0.36% | 16.67% | 0.039484 |
|  | CAY69995 | Peptide methionine sulfoxide reductase, reverses the oxidation of methionine residues | 0.36% | 16.67% | 0.039484 |
